# Crystal structure of *N*-(2-benzoyl-5-ethynylphen­yl)quinoline-2-carboxamide

**DOI:** 10.1107/S2056989017004601

**Published:** 2017-03-28

**Authors:** Diana Peña-Solórzano, Burkhard König, Cesar A. Sierra, Cristian Ochoa-Puentes

**Affiliations:** a Grupo de Investigación en Macromoléculas, Departamento de Química, Universidad Nacional de Colombia-Sede Bogotá, A.A. 5997 Bogotá, Colombia; b Institute of Organic Chemistry, University of Regensburg, 93040 Regensburg, Germany

**Keywords:** crystal structure, quinoline, benzoyl-5-ethynylphen­yl, hydrogen bonding

## Abstract

In the title compound, C_25_H_16_N_2_O_2_, the quinoline ring system is essentially planar, with a maximum deviation of 0.030 (1) Å, and forms a dihedral angle of 20.9 (1)° with benzoyl benzene ring. The unsubstituted phenyl ring forms dihedral angles of 52.7 (1)° with the quinoline ring system and 54.1 (1)° with the ethynyl-substituted benzene ring. The mol­ecule contains an intra­molecular bifurcated N—H⋯(O,N) hydrogen bond, forming *S*(5) and *S*(6) rings, which may influence the conformation of the mol­ecule. In the crystal, weak C—H⋯O hydrogen bonds link the mol­ecules into a three-dimensional network. In addition, the three-dimensional structure contains π–π stacking inter­actions, with centroid–centroid distances of 3.695 (1) and 3.751 (1) Å.

## Abstract 2

In the title compound, the quinoline ring system forms a dihedral angle of 20.9 (1)° with ethynyl-substituted benzene ring. The unsubstituted phenyl ring forms a dihedral angles of 52.7 (1)° with the quinoline ring system and 54.1 (1)° with the ethynyl-substituted benzene ring. An intra­molecular bifurcated N—H⋯(O,N) hydrogen bond forms *S*(5) and *S*(6) rings. In the crystal, weak C—H⋯O hydrogen bonds link the mol­ecules into a three-dimensional network.

## Chemical context   

Benzo­phenones are inter­mediates for the synthesis of pharmaceutical and bioactive materials and are used extensively in the field of medicinal chemistry. The biological activity of these ligands can be attributed to distinct chemical and biochemical advantages: they are chemically more stable than diazo esters, aryl azides and diazirines, and can be manipulated in ambient light and can be activated at 350–360 nm, avoiding protein-damaging wavelengths. These properties produce highly efficient covalent modifications of macromolecules, frequently with remarkable specificity (Dormán & Prestwich, 1994 ▸). Several benzo­phenones are used in industry, cosmetics, medicine and agriculture (Sweetman *et al.*, 2007 ▸), and their role as potential anti­cancer agents and anti­biotics has also been examined. In addition, research has been performed on the use of benzo­phenones as modulators of GABAA receptors (Kopanitsa *et al.*, 2002 ▸), COX-1/COX-2 inhibitors (Dannhardt *et al.*, 2002 ▸) and EGFR/erbB2 dual inhibitors (Zhang *et al.*, 2004 ▸).
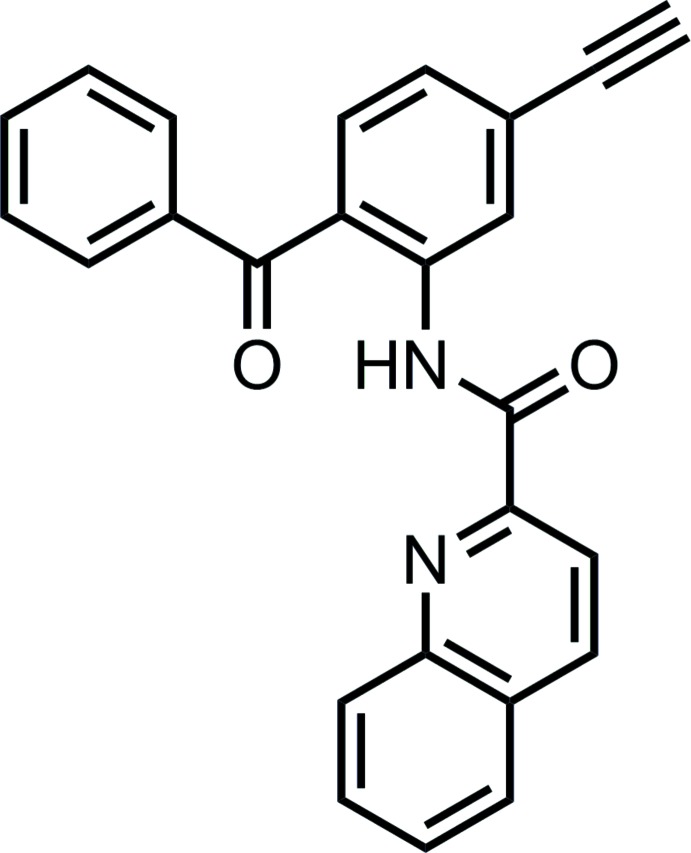


## Structural commentary   

**Figure 1:**
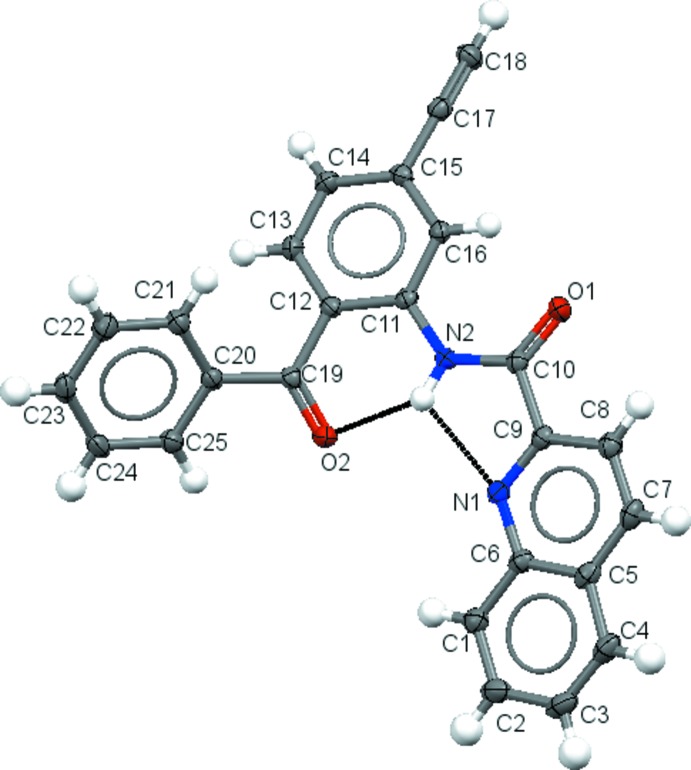
The mol­ecular structure of the title compound, with displacement ellipsoids drawn at the 50% probability level. Hydrogen bonds are shown as dotted lines.

The mol­ecular structure of the title compound is shown in Fig. 1 ▸. The quinoline ring system (C1–C9/N1) is essentially planar, with a maximum deviation of 0.030 (1) for C8 and forms a dihedral angle of 20.9 (1)° with ethynyl-substituted benzene ring (C11–C16). The benzoyl ring (C20–C25) forms dihedral angles of 52.7 (1)° with the quinoline ring system and 54.1 (1)° with the ethynyl-substituted benzene ring. The mol­ecule contains an intra­molecular bifurcated N—H⋯(N,O) hydrogen bond (see Table 1 ▸), forming *S*(5) and *S*(6) rings, which may influence the conformation of the mol­ecule.

## Supra­molecular features   

**Figure 2:**
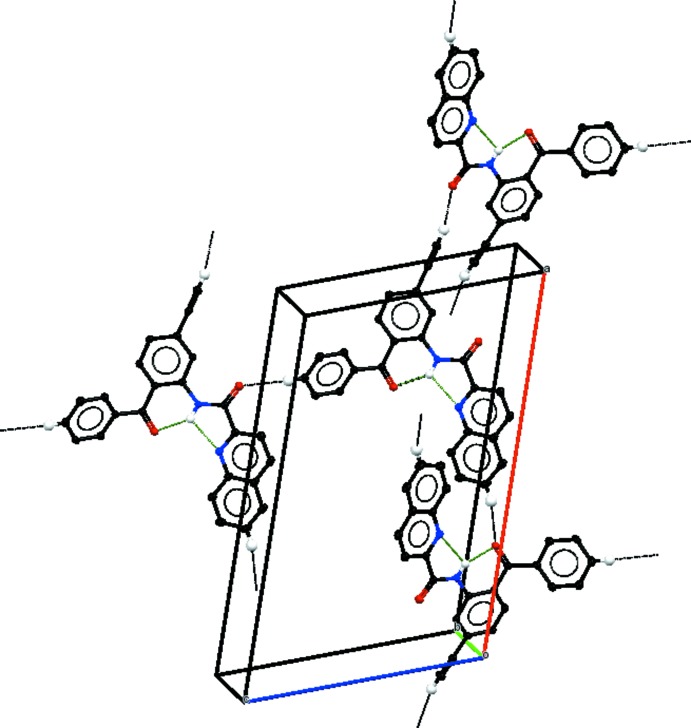
A partial packing diagram of the title compound, viewed approximately along the *b* axis, with inter­molecular hydrogen bonds shown as black dotted lines and intra­molecular hydrogen bonds shown as green dotted lines.

In the crystal, weak C—H⋯O hydrogen bonds (Table 1 ▸, Fig. 2 ▸) link the mol­ecules into a three-dimensional network. In addition, the three-dimensional structure contains π–π stacking inter­actions with centroid–centroid distances of 3.695 (1) Å for *Cg*1⋯*Cg*2(*x*, 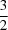 − *y*, −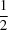 + *z*) and 3.751 (1) Å for *Cg*3⋯*Cg*3(1 − *x*, 1 − *y*, −*z*) where *Cg*1, *Cg*2 and *Cg*3 are the centroids of the C11–C16, C20–C25 and C1–C6 rings, respectively.

## Database survey   

A search of the Cambridge Structural Database (Groom *et al.*, 2016 ▸; Version 1.18, April 2016) revealed 12 related structures. There are three reports for (4-ethynylphen­yl)(phen­yl)methanone derivatives with different substituents (Szafert *et al.* 2008 ▸, 2012 ▸; Khera *et al.* 2012 ▸). There are two reports where *N*-(2-benzoyl­phen­yl)quinoline-2-carboxamide moieties are reported (Maurizot *et al.* 2004 ▸; Hu *et al.* 2009 ▸) and seven reports for 3-ethynylaniline derivatives (Li *et al.* 2012 ▸; Cummings *et al.* 2010 ▸; Khan *et al.* 2003 ▸; Dominguez *et al.* 2003 ▸; Wang *et al.* 2003 ▸; Yi *et al.* 2008 ▸; Armitt *et al.* 2008 ▸).

## Synthesis and crystallization   

**Figure 3:**
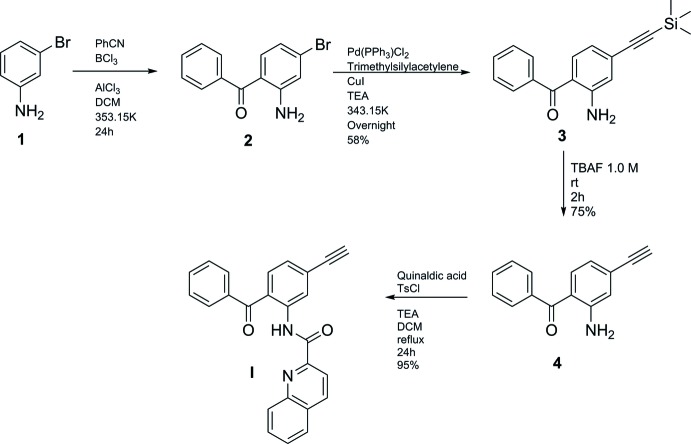
The reaction scheme for the synthesis of the title compound.

The title compound was prepared using 3-bromo­aniline (**1**, Fig. 3 ▸) as starting reagent in the presence of boron trichloride (1.1 equiv), AlCl_3_ (1.1 equiv) and benzo­nitrile (3 equiv) for 24 h at approximately 353 K. The solution was extracted with DCM, dried and concentrated to obtain (2-amino-4-bromo­phen­yl)(phen­yl)methanone (**2**) (petroleum ether:ethyl acetate 9:1, 0.52). Compound **2** (1.8 mmol) was dissolved in tri­ethyl­amine, Pd(PPh_3_)_2_Cl_2_ (0.05 eq), tri­methyl­silyl­acetyl­ene (1.5 eq) and copper iodine (0.1 eq) were added and the solution was heated to approximately 343 K overnight. The organic phase was separated and concentrated (petroleum ether:ethyl acetate 7:1, 0.70) and the fraction containing the product (75%) was collected and used for the next step. A solution of compound **3** (0.4 mol, 1 eq) in tetra­hydro­furane was stirred and cooled in an ice bath, tetra-*n*-butyl­ammonium fluoride (1.5 eq) was added and the reaction was stirred for two hours. The organic layer was separated and dried over magnesium sulfate to obtain compound **4** (petroleum ether:ethyl acetate 7:1, 0.60). The title compound (I) (Fig. 3 ▸) was prepared by refluxing a mixture of quinaldic acid, tri­ethyl­amine, *p*-toluene­sulfonyl chloride and compound **4** for 24 h in di­chloro­methane. After evaporation of the CH_2_Cl_2_, the compound was purified by silica column chromatography (petroleum ether:ethyl acetate 7:1, 0.36). Single colourless block-shaped crystals of (I) were obtained by slow evaporation in di­chloro­methane in a closed flask with petroleum ether.

***N*-(2-benzoyl-5-ethynylphen­yl)quinoline-2-carboxamide (I):** Colourless solid (0.323 g, 95%, PE:EA 7:1, *R*_f_ = 0.36). **^1^H NMR (400 MHz, CDCl_3_)**: δ 9.11 (*d*, *^3^J* = 1.4 Hz, 1H), 8.41 (*m*, 3H), 7.89 (*d*, *^3^J* = 8.2 Hz, 1H), 7.84 (*m*, 3H), 7.67 (*m*, 1H), 7.60 (*m*, 2H), 7.50 (*dd*, *^3^J* = 10.4, *^3^J* = 4.6 Hz, 2H), 7.28 (*m*, 1H), 3.27 (*s*, 1H, CCH). **^13^C NMR (100 MHz, CDCl_3_)**: δ 198.0 (C_quat_), 163.7 (C_quat_), 149.6 (C_quat_), 146.6 (C_quat_), 139.7 (C_quat_), 138.6 (C_quat_), 137.6 (C_quat_), 133.1 (+), 132.5 (+), 130.5 (+), 130.2 (+), 129.9 (+), 129.4 (+), 128.3 (+), 127.6 (+), 125.8 (+), 124.8 (+), 118.4 (+), 82.8 (C_quat_), 80.3 (+).

## Refinement   

Crystal data, data collection and structure refinement details are summarized in Table 2 ▸. All non-hydrogen atoms were refined anisotropically. Hydrogen-atom positions were calculated geometrically and refined using the riding model: N—H = 0.86 Å and C—H = 0.93 Å with *U*_iso_(H) = 1.2*U*_eq_(C,N).

## Supplementary Material

Crystal structure: contains datablock(s) I. DOI: 10.1107/S2056989017004601/lh5838sup1.cif

Structure factors: contains datablock(s) I. DOI: 10.1107/S2056989017004601/lh5838Isup2.hkl

Supporting information file. DOI: 10.1107/S2056989017004601/lh5838Isup3.cml

CCDC reference: 1539719

Additional supporting information:  crystallographic information; 3D view; checkCIF report

## Figures and Tables

**Table 1:** Hydrogen-bond geometry (Å, °)

*D*—H⋯*A*	*D*—H	H⋯*A*	*D*⋯*A*	*D*—H⋯*A*
N2—H2⋯O2	0.86	2.03	2.701 (12)	135
N2—H2⋯N1	0.86	2.24	2.658 (13)	110
C3—H3⋯O2^i^	0.93	2.47	3.346 (16)	158
C18—H18⋯O1^ii^	0.93	2.33	3.242 (15)	167
C23—H23⋯O1^iii^	0.93	2.56	3.476 (14)	168

Symmetry codes: (i) 
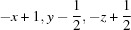
; (ii) 
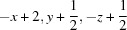
; (iii) 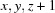.

**Table 2:** Experimental details

Crystal data	
Chemical formula	C_25_H_16_N_2_O_2_
*M*_r_	376.40
Crystal system, space group	Monoclinic, *P*2_1_/*c*
Temperature (K)	123
*a*, *b*, *c* (Å)	20.2686 (3), 7.58016 (11), 12.6109 (2)
β (°)	107.6002 (17)
*V* (Å^3^)	1846.84 (5)
*Z*	4
Radiation type	Cu *K*α
μ (mm^−1^)	0.70
Crystal size (mm)	0.20 × 0.12 × 0.08

Data collection	
Diffractometer	Rigaku Oxfor Diffraction SuperNova, Single source at offset, Atlas
Absorption correction	Analytical (*CrysAlis PRO*; Rigaku Oxford Diffraction, 2015 ▸)
*T*_min_, *T*_max_	0.923, 0.964
No. of measured, independent and observed [*I* > 2σ(*I*)] reflections	14681, 3484, 3170
*R*_int_	0.020
(sin θ/λ)_max_ (Å^−1^)	0.612

Refinement	
*R*[*F* ^2^ > 2σ(*F* ^2^)], *wR*(*F* ^2^), *S*	0.034, 0.092, 1.05
No. of reflections	3484
No. of parameters	262
H-atom treatment	H-atom parameters constrained
Δρ_max_, Δρ_min_ (e Å^−3^)	0.21, −0.25

Computer programs: *CrysAlis PRO* (Rigaku Oxford Diffraction, 2015 ▸), *SHELXT2014* (Sheldrick, 2015*a* ▸), *SHELXL* (Sheldrick, 2015*b* ▸) and *OLEX2* (Dolomanov *et al.*, 2009 ▸).

## supplementary crystallographic information

### Crystal data

**Table:**

C_25_H_16_N_2_O_2_	*F*(000) = 784
*M**_r_* = 376.40	*D*_x_ = 1.354 Mg m^−^^3^
Monoclinic, *P*2_1_/*c*	Cu *K*α radiation, λ = 1.54184 Å
*a* = 20.2686 (3) Å	Cell parameters from 8847 reflections
*b* = 7.58016 (11) Å	θ = 4.6–70.5°
*c* = 12.6109 (2) Å	µ = 0.70 mm^−^^1^
β = 107.6002 (17)°	*T* = 123 K
*V* = 1846.84 (5) Å^3^	Block, colourless
*Z* = 4	0.20 × 0.12 × 0.08 mm

### Data collection

**Table:**

Rigaku Oxfor Diffraction SuperNova, Single source at offset, Atlas diffractometer	3484 independent reflections
Radiation source: micro-focus sealed X-ray tube, SuperNova (Cu) X-ray Source	3170 reflections with *I* > 2σ(*I*)
Mirror monochromator	*R*_int_ = 0.020
Detector resolution: 5.1773 pixels mm^-1^	θ_max_ = 70.6°, θ_min_ = 4.6°
ω scans	*h* = −24→24
Absorption correction: analytical (CrysAlis PRO; Rigaku Oxford Diffraction, 2015)	*k* = −9→8
*T*_min_ = 0.923, *T*_max_ = 0.964	*l* = −15→15
14681 measured reflections	

### Refinement

**Table:**

Refinement on *F*^2^	Hydrogen site location: inferred from neighbouring sites
*R*[*F*^2^ > 2σ(*F*^2^)] = 0.034	H-atom parameters constrained
*wR*(*F*^2^) = 0.092	*w* = 1/[σ^2^(*F*_o_^2^) + (0.0466*P*)^2^ + 0.6041*P*] where *P* = (*F*_o_^2^ + 2*F*_c_^2^)/3
*S* = 1.05	(Δ/σ)_max_ = 0.001
3484 reflections	Δρ_max_ = 0.21 e Å^−^^3^
262 parameters	Δρ_min_ = −0.25 e Å^−^^3^
0 restraints	

### Special details

**Table:**

Geometry. All esds (except the esd in the dihedral angle between two l.s. planes) are estimated using the full covariance matrix. The cell esds are taken into account individually in the estimation of esds in distances, angles and torsion angles; correlations between esds in cell parameters are only used when they are defined by crystal symmetry. An approximate (isotropic) treatment of cell esds is used for estimating esds involving l.s. planes.

### Fractional atomic coordinates and isotropic or equivalent isotropic displacement parameters (Å^2^)

**Table:**

	*x*	*y*	*z*	*U*_iso_*/*U*_eq_	
O1	0.80756 (4)	0.44245 (12)	0.17559 (6)	0.0251 (2)	
O2	0.71578 (4)	0.59252 (12)	0.48953 (6)	0.0252 (2)	
N2	0.77463 (5)	0.50473 (13)	0.33109 (7)	0.0187 (2)	
H2	0.7400	0.4943	0.3565	0.022*	
N1	0.64362 (5)	0.43688 (13)	0.21772 (8)	0.0204 (2)	
C11	0.83488 (5)	0.57608 (14)	0.40482 (9)	0.0176 (2)	
C12	0.83608 (5)	0.61666 (15)	0.51504 (9)	0.0185 (2)	
C10	0.76382 (6)	0.45000 (15)	0.22485 (9)	0.0192 (2)	
C15	0.95056 (5)	0.69688 (15)	0.44286 (9)	0.0196 (2)	
C16	0.89330 (5)	0.61127 (15)	0.37169 (9)	0.0188 (2)	
H16	0.8940	0.5772	0.3012	0.023*	
C20	0.78417 (6)	0.55345 (15)	0.67512 (9)	0.0195 (2)	
C9	0.69003 (6)	0.39498 (15)	0.16801 (9)	0.0197 (2)	
C19	0.77474 (6)	0.58849 (15)	0.55491 (9)	0.0195 (2)	
C6	0.57653 (6)	0.38875 (15)	0.16679 (9)	0.0214 (2)	
C13	0.89513 (6)	0.69693 (16)	0.58579 (9)	0.0214 (2)	
H13	0.8965	0.7225	0.6585	0.026*	
C14	0.95156 (6)	0.73953 (16)	0.55107 (9)	0.0224 (2)	
H14	0.9897	0.7959	0.5991	0.027*	
C25	0.73004 (6)	0.59635 (16)	0.71778 (10)	0.0234 (3)	
H25	0.6904	0.6510	0.6726	0.028*	
C5	0.55645 (6)	0.29271 (16)	0.06529 (9)	0.0244 (3)	
C21	0.84242 (6)	0.46767 (16)	0.74319 (9)	0.0231 (3)	
H21	0.8786	0.4383	0.7154	0.028*	
C1	0.52563 (6)	0.43856 (17)	0.21699 (10)	0.0259 (3)	
H1	0.5383	0.5024	0.2830	0.031*	
C8	0.67500 (6)	0.30330 (16)	0.06614 (10)	0.0244 (3)	
H8	0.7096	0.2795	0.0338	0.029*	
C23	0.79376 (6)	0.47211 (18)	0.89465 (10)	0.0281 (3)	
H23	0.7972	0.4462	0.9682	0.034*	
C22	0.84671 (6)	0.42576 (17)	0.85222 (10)	0.0268 (3)	
H22	0.8853	0.3664	0.8968	0.032*	
C4	0.48571 (6)	0.24644 (17)	0.01817 (11)	0.0302 (3)	
H4	0.4719	0.1823	−0.0477	0.036*	
C24	0.73547 (6)	0.55729 (18)	0.82745 (10)	0.0276 (3)	
H24	0.6999	0.5883	0.8562	0.033*	
C7	0.60855 (6)	0.25045 (17)	0.01627 (10)	0.0274 (3)	
H7	0.5976	0.1868	−0.0498	0.033*	
C2	0.45806 (6)	0.39319 (19)	0.16885 (11)	0.0319 (3)	
H2A	0.4250	0.4271	0.2023	0.038*	
C3	0.43768 (6)	0.29527 (19)	0.06876 (11)	0.0337 (3)	
H3	0.3915	0.2641	0.0373	0.040*	
C17	1.00703 (6)	0.75029 (16)	0.40188 (9)	0.0220 (2)	
C18	1.05212 (6)	0.80433 (17)	0.37023 (10)	0.0274 (3)	
H18	1.0878	0.8471	0.3452	0.033*	

### Atomic displacement parameters (Å^2^)

**Table:**

	*U*^11^	*U*^22^	*U*^33^	*U*^12^	*U*^13^	*U*^23^
O1	0.0219 (4)	0.0344 (5)	0.0207 (4)	−0.0005 (3)	0.0087 (3)	−0.0044 (3)
O2	0.0171 (4)	0.0381 (5)	0.0206 (4)	0.0014 (3)	0.0060 (3)	0.0014 (3)
N2	0.0165 (4)	0.0236 (5)	0.0170 (4)	−0.0014 (4)	0.0064 (3)	−0.0008 (4)
N1	0.0194 (5)	0.0217 (5)	0.0190 (5)	−0.0017 (4)	0.0041 (4)	0.0008 (4)
C11	0.0170 (5)	0.0171 (5)	0.0179 (5)	0.0015 (4)	0.0041 (4)	0.0014 (4)
C12	0.0178 (5)	0.0205 (6)	0.0179 (5)	0.0017 (4)	0.0063 (4)	0.0016 (4)
C10	0.0202 (5)	0.0186 (5)	0.0186 (5)	0.0011 (4)	0.0055 (4)	0.0005 (4)
C15	0.0172 (5)	0.0210 (6)	0.0217 (5)	0.0014 (4)	0.0075 (4)	0.0018 (4)
C16	0.0194 (5)	0.0210 (6)	0.0165 (5)	0.0011 (4)	0.0063 (4)	0.0010 (4)
C20	0.0199 (5)	0.0208 (6)	0.0191 (5)	−0.0039 (4)	0.0077 (4)	−0.0033 (4)
C9	0.0208 (5)	0.0187 (5)	0.0186 (5)	0.0006 (4)	0.0043 (4)	0.0015 (4)
C19	0.0189 (5)	0.0199 (6)	0.0202 (5)	0.0004 (4)	0.0067 (4)	−0.0018 (4)
C6	0.0201 (5)	0.0208 (6)	0.0211 (5)	−0.0016 (4)	0.0029 (4)	0.0042 (4)
C13	0.0214 (5)	0.0263 (6)	0.0167 (5)	−0.0004 (4)	0.0061 (4)	−0.0024 (4)
C14	0.0184 (5)	0.0260 (6)	0.0215 (5)	−0.0028 (4)	0.0042 (4)	−0.0028 (5)
C25	0.0199 (5)	0.0284 (6)	0.0226 (6)	−0.0028 (5)	0.0077 (4)	−0.0042 (5)
C5	0.0252 (6)	0.0218 (6)	0.0219 (6)	−0.0031 (5)	0.0008 (5)	0.0028 (5)
C21	0.0228 (5)	0.0257 (6)	0.0220 (6)	−0.0003 (5)	0.0087 (4)	−0.0013 (5)
C1	0.0232 (6)	0.0305 (7)	0.0229 (6)	−0.0013 (5)	0.0053 (5)	0.0036 (5)
C8	0.0269 (6)	0.0245 (6)	0.0215 (6)	0.0009 (5)	0.0071 (5)	−0.0022 (5)
C23	0.0329 (6)	0.0345 (7)	0.0179 (5)	−0.0118 (5)	0.0093 (5)	−0.0020 (5)
C22	0.0281 (6)	0.0292 (6)	0.0211 (6)	−0.0022 (5)	0.0042 (5)	0.0020 (5)
C4	0.0283 (6)	0.0300 (7)	0.0255 (6)	−0.0077 (5)	−0.0020 (5)	0.0010 (5)
C24	0.0250 (6)	0.0376 (7)	0.0247 (6)	−0.0076 (5)	0.0144 (5)	−0.0079 (5)
C7	0.0321 (6)	0.0253 (6)	0.0210 (6)	−0.0031 (5)	0.0025 (5)	−0.0049 (5)
C2	0.0206 (6)	0.0410 (8)	0.0335 (7)	−0.0012 (5)	0.0073 (5)	0.0082 (6)
C3	0.0212 (6)	0.0391 (8)	0.0341 (7)	−0.0080 (5)	−0.0017 (5)	0.0083 (6)
C17	0.0203 (5)	0.0231 (6)	0.0206 (5)	−0.0006 (4)	0.0034 (4)	−0.0037 (4)
C18	0.0260 (6)	0.0315 (7)	0.0282 (6)	−0.0048 (5)	0.0134 (5)	−0.0042 (5)

### Geometric parameters (Å, º)

**Table:**

O1—C10	1.2282 (14)	C14—H14	0.9300
O2—C19	1.2301 (14)	C25—H25	0.9300
N2—H2	0.8600	C25—C24	1.3858 (17)
N2—C11	1.4006 (14)	C5—C4	1.4200 (16)
N2—C10	1.3555 (14)	C5—C7	1.4124 (17)
N1—C9	1.3174 (15)	C21—H21	0.9300
N1—C6	1.3661 (14)	C21—C22	1.3881 (16)
C11—C12	1.4167 (15)	C1—H1	0.9300
C11—C16	1.3952 (15)	C1—C2	1.3637 (17)
C12—C19	1.4907 (15)	C8—H8	0.9300
C12—C13	1.3975 (16)	C8—C7	1.3630 (17)
C10—C9	1.5087 (15)	C23—H23	0.9300
C15—C16	1.3942 (15)	C23—C22	1.3816 (18)
C15—C14	1.3965 (16)	C23—C24	1.3876 (18)
C15—C17	1.4487 (15)	C22—H22	0.9300
C16—H16	0.9300	C4—H4	0.9300
C20—C19	1.4932 (15)	C4—C3	1.367 (2)
C20—C25	1.3984 (15)	C24—H24	0.9300
C20—C21	1.3932 (16)	C7—H7	0.9300
C9—C8	1.4104 (16)	C2—H2A	0.9300
C6—C5	1.4209 (17)	C2—C3	1.414 (2)
C6—C1	1.4152 (17)	C3—H3	0.9300
C13—H13	0.9300	C17—C18	1.1759 (17)
C13—C14	1.3810 (16)	C18—H18	0.9300
			
C11—N2—H2	115.8	C24—C25—C20	119.96 (11)
C10—N2—H2	115.8	C24—C25—H25	120.0
C10—N2—C11	128.49 (9)	C4—C5—C6	118.79 (11)
C9—N1—C6	117.66 (10)	C7—C5—C6	117.52 (10)
N2—C11—C12	119.21 (9)	C7—C5—C4	123.69 (11)
C16—C11—N2	121.57 (10)	C20—C21—H21	119.8
C16—C11—C12	119.21 (10)	C22—C21—C20	120.31 (11)
C11—C12—C19	122.09 (10)	C22—C21—H21	119.8
C13—C12—C11	118.56 (10)	C6—C1—H1	119.9
C13—C12—C19	119.19 (10)	C2—C1—C6	120.23 (12)
O1—C10—N2	126.05 (10)	C2—C1—H1	119.9
O1—C10—C9	120.68 (10)	C9—C8—H8	120.8
N2—C10—C9	113.27 (9)	C7—C8—C9	118.44 (11)
C16—C15—C14	120.01 (10)	C7—C8—H8	120.8
C16—C15—C17	119.61 (10)	C22—C23—H23	120.0
C14—C15—C17	120.28 (10)	C22—C23—C24	119.96 (11)
C11—C16—H16	119.6	C24—C23—H23	120.0
C15—C16—C11	120.85 (10)	C21—C22—H22	119.9
C15—C16—H16	119.6	C23—C22—C21	120.14 (11)
C25—C20—C19	118.34 (10)	C23—C22—H22	119.9
C21—C20—C19	122.21 (10)	C5—C4—H4	119.7
C21—C20—C25	119.26 (10)	C3—C4—C5	120.62 (12)
N1—C9—C10	117.07 (10)	C3—C4—H4	119.7
N1—C9—C8	124.31 (10)	C25—C24—C23	120.33 (11)
C8—C9—C10	118.62 (10)	C25—C24—H24	119.8
O2—C19—C12	120.71 (10)	C23—C24—H24	119.8
O2—C19—C20	119.02 (10)	C5—C7—H7	120.1
C12—C19—C20	120.27 (9)	C8—C7—C5	119.78 (11)
N1—C6—C5	122.26 (11)	C8—C7—H7	120.1
N1—C6—C1	118.36 (10)	C1—C2—H2A	119.6
C1—C6—C5	119.38 (10)	C1—C2—C3	120.85 (12)
C12—C13—H13	119.0	C3—C2—H2A	119.6
C14—C13—C12	122.08 (10)	C4—C3—C2	120.12 (11)
C14—C13—H13	119.0	C4—C3—H3	119.9
C15—C14—H14	120.4	C2—C3—H3	119.9
C13—C14—C15	119.14 (10)	C18—C17—C15	175.81 (13)
C13—C14—H14	120.4	C17—C18—H18	180.0
C20—C25—H25	120.0		
			
O1—C10—C9—N1	−167.73 (11)	C19—C12—C13—C14	−174.75 (11)
O1—C10—C9—C8	12.16 (17)	C19—C20—C25—C24	176.64 (11)
N2—C11—C12—C19	−0.98 (16)	C19—C20—C21—C22	−175.07 (11)
N2—C11—C12—C13	−176.45 (10)	C6—N1—C9—C10	179.62 (9)
N2—C11—C16—C15	174.15 (10)	C6—N1—C9—C8	−0.27 (17)
N2—C10—C9—N1	12.51 (15)	C6—C5—C4—C3	0.81 (18)
N2—C10—C9—C8	−167.60 (10)	C6—C5—C7—C8	−0.59 (18)
N1—C9—C8—C7	−1.52 (18)	C6—C1—C2—C3	0.3 (2)
N1—C6—C5—C4	179.47 (11)	C13—C12—C19—O2	148.36 (11)
N1—C6—C5—C7	−1.25 (17)	C13—C12—C19—C20	−32.13 (16)
N1—C6—C1—C2	179.99 (11)	C14—C15—C16—C11	3.68 (17)
C11—N2—C10—O1	5.23 (19)	C25—C20—C19—O2	−25.56 (16)
C11—N2—C10—C9	−175.02 (10)	C25—C20—C19—C12	154.92 (11)
C11—C12—C19—O2	−27.08 (17)	C25—C20—C21—C22	−0.18 (17)
C11—C12—C19—C20	152.43 (11)	C5—C6—C1—C2	0.63 (18)
C11—C12—C13—C14	0.85 (17)	C5—C4—C3—C2	0.2 (2)
C12—C11—C16—C15	−4.60 (16)	C21—C20—C19—O2	149.37 (11)
C12—C13—C14—C15	−1.80 (18)	C21—C20—C19—C12	−30.15 (16)
C10—N2—C11—C12	−176.82 (11)	C21—C20—C25—C24	1.56 (17)
C10—N2—C11—C16	4.43 (18)	C1—C6—C5—C4	−1.20 (17)
C10—C9—C8—C7	178.59 (11)	C1—C6—C5—C7	178.08 (11)
C16—C11—C12—C19	177.81 (10)	C1—C2—C3—C4	−0.7 (2)
C16—C11—C12—C13	2.34 (16)	C22—C23—C24—C25	−0.03 (19)
C16—C15—C14—C13	−0.46 (17)	C4—C5—C7—C8	178.64 (12)
C20—C25—C24—C23	−1.46 (19)	C24—C23—C22—C21	1.41 (19)
C20—C21—C22—C23	−1.30 (19)	C7—C5—C4—C3	−178.42 (12)
C9—N1—C6—C5	1.67 (16)	C17—C15—C16—C11	−172.73 (10)
C9—N1—C6—C1	−177.67 (10)	C17—C15—C14—C13	175.94 (11)
C9—C8—C7—C5	1.89 (18)		

### Hydrogen-bond geometry (Å, º)

**Table:**

*D*—H···*A*	*D*—H	H···*A*	*D*···*A*	*D*—H···*A*
N2—H2···O2	0.86	2.03	2.701 (12)	135
N2—H2···N1	0.86	2.24	2.658 (13)	110
C3—H3···O2^i^	0.93	2.47	3.346 (16)	158
C18—H18···O1^ii^	0.93	2.33	3.242 (15)	167
C23—H23···O1^iii^	0.93	2.56	3.476 (14)	168

Symmetry codes: (i) −*x*+1, *y*−1/2, −*z*+1/2; (ii) −*x*+2, *y*+1/2, −*z*+1/2; (iii) *x*, *y*, *z*+1.
